# A Series of Supramolecular Complexes for Solar Energy Conversion via Water Reduction to Produce Hydrogen: An Excited State Kinetic Analysis of Ru(II),Rh(III),Ru(II) Photoinitiated Electron Collectors

**DOI:** 10.3390/ma5010027

**Published:** 2011-12-27

**Authors:** Travis A. White, Jessica D. Knoll, Shamindri M. Arachchige, Karen J. Brewer

**Affiliations:** Department of Chemistry, Virginia Tech, Blacksburg, VA 24061-0212, USA; E-Mails: whiteta@vt.edu (T.A.W.); jdknoll@vt.edu (J.D.K.); arachsm@vt.edu (S.M.A.)

**Keywords:** intramolecular electron transfer, photoinitiated electron collection, supramolecular photocatalysis, excited state quenching, hydrogen production

## Abstract

Mixed-metal supramolecular complexes have been designed that photochemically absorb solar light, undergo photoinitiated electron collection and reduce water to produce hydrogen fuel using low energy visible light. This manuscript describes these systems with an analysis of the photophysics of a series of six supramolecular complexes, [{(TL)_2_Ru(dpp)}_2_RhX_2_](PF_6_)_5_ with TL = bpy, phen or Ph_2_phen with X = Cl or Br. The process of light conversion to a fuel requires a system to perform a number of complicated steps including the absorption of light, the generation of charge separation on a molecular level, the reduction by one and then two electrons and the interaction with the water substrate to produce hydrogen. The manuscript explores the rate of intramolecular electron transfer, rate of quenching of the supramolecules by the DMA electron donor, rate of reduction of the complex by DMA from the ^3^MLCT excited state, as well as overall rate of reduction of the complex via visible light excitation. Probing a series of complexes in detail exploring the variation of rates of important reactions as a function of sub-unit modification provides insight into the role of each process in the overall efficiency of water reduction to produce hydrogen. The kinetic analysis shows that the complexes display different rates of excited state reactions that vary with TL and halide. The role of the MLCT excited state is elucidated by this kinetic study which shows that the ^3^MLCT state and not the ^3^MMCT is likely that key contributor to the photoreduction of these complexes. The kinetic analysis of the excited state dynamics and reactions of the complexes are important as this class of supramolecules behaves as photoinitiated electron collectors and photocatalysts for the reduction of water to hydrogen.

## 1. Introduction

The demand for alternative fuel sources is continually increasing. An attractive approach to this issue is the conversion of solar energy to chemical energy in the form of H_2_O splitting to produce H_2_ fuel [1,2]. At neutral pH and 25 °C, H_2_O can be split into H_2_ and O_2_ via a multi-electron pathway that requires 1.23 V [3]. Sunlight provides an abundant amount of energy to the Earth’s surface that contains the required energy to drive this thermodynamically uphill, multi-electron reaction. However, H_2_O does not absorb an appreciable amount of sunlight reaching the surface, therefore systems must be designed to efficiently absorb light and deliver appropriate charges to H_2_O. One means of achieving this goal is through the use of supramolecular complexes [4]. In this arena, supramolecular complexes are described as molecular machines comprised of multiple molecular components whose individual properties contribute to the overall functioning of the system [5]. Supramolecular complexes of interest in solar energy conversion schemes are photochemical molecular devices (PMDs) as they perform a specific light-driven task utilizing solar energy as the thermodynamic driving force for a desired chemical reaction. Engineering PMDs to perform specific, complex functions at the molecular level allows for the exploitation of these systems as potential photocatalysts. Systems can be designed to perform photoinduced vectoral electron transfer and charge migration between appropriate electron donor (ED), such as an electron rich, metal-based light absorber (LA), and electron acceptor (EA) sites. Generating this photoinduced charge separation and migration within PMDs is of considerable interest in the realm of solar energy conversion schemes [5].

An application of PMDs is the generation of multielectron photocatalysts that utilize photoinduced processes to deliver multiple reducing equivalents to a central site which may interact with an appropriate substrate. Photoinitiated electron collectors (PECs) are a type of PMD typically comprised of metal-based LA subunits covalently bound to an electron collecting (EC) site through polyazine bridging ligands (BL) [4,6]. Varying the molecular components within the PEC such as polypyridyl terminal ligands (TL) and polyazine BLs attached to the LA modulates the photoactive and redox-active properties of the PECs. The first reported PMD for PEC, [{(bpy)_2_Ru(dpb)}_2_IrCl_2_](PF_6_)_5_ (bpy = 2,2′-bipyridine; dpb = 2,3-bis(2-pyridyl)benzoquinoxaline) undergoes electron collection on the dpb BL π* LUMO upon visible light excitation [6]. The homobimetallic complexes [(phen)_2_Ru(BL)Ru(phen)_2_](PF_6_)_4_ collect up to two or four electrons on the BL (π*) orbitals (phen = 1,10-phenanthroline; BL = 9,11,20,22-tetraazatetrapyrido[3,2-a:2′3′-c:3′′,2′′-1:2′′′,3′′′-n]pentacene (tatpp) or 9,11,20,22-tetraazatetrapyrido[3,2-a:2′3′-c:3′′,2′′-1:2′′′,3′′′-n]pentacene-10,21-quinone (tatpq)) [7,8]. The Ru monometallic [(bpy)_2_Ru(pbn)](PF_6_)_2_ undergoes proton-coupled two-electron reduction of the NAD/NADH^+^ model ligand to produce [(bpy)_2_Ru(pbnHH)]^2+^ (pbn = 2-(2-pyridyl)benzo[*b*]-1,5-naphthyridine, NAD = nicotinamide adenine dinucleotide) [9,10]. These early systems display ligand-centered photoinitiated electron collection, but do not perform photocatalytic reduction of H_2_O to H_2_.

**Figure 1:**
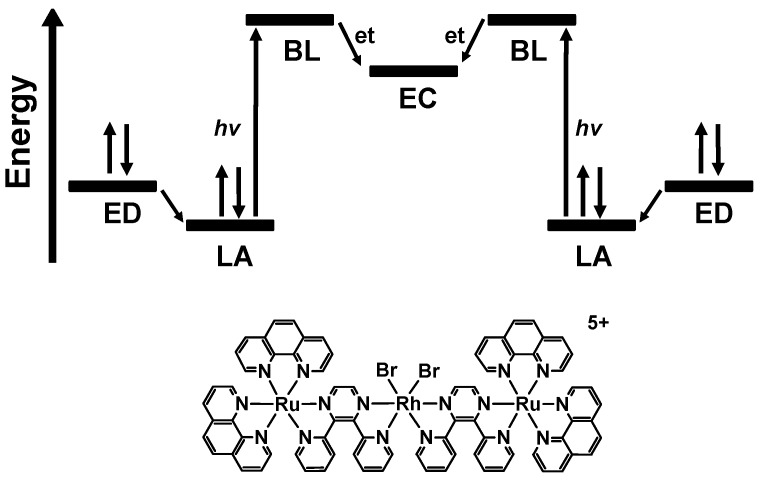
Photoinitiated electron collection at a central site using the ED-LA-BL-EC-BL-LA-ED structural motif (ED = sacrificial electron donor; LA = light absorber; BL = bridging ligand; EC = electron collector; et = intramolecular electron transfer). Structure for the Ru(II),Rh(III),Ru(II) supramolecular complex [{(phen)_2_Ru(dpp)}_2_RhBr_2_](PF_6_)_5_ is also shown (phen = 1,10-phenanthroline; dpp = 2,3-bis(2-pyridyl)pyrazine).

Modifying the [{(bpy)_2_Ru(dpb)}_2_IrCl_2_](PF_6_)_5_ trimetallic by changing the BL from dpb to dpp and the central metal from Ir(III) to Rh(III) generates [{(bpy)_2_Ru(dpp)}_2_RhCl_2_](PF_6_)_5_ (dpp = 2,3-bis(2-pyridyl)pyrazine) [11]. This Ru(II),Rh(III),Ru(II) trimetallic complex displays orbital inversion with the LUMO now localized on the Rh(III) metal center and is the first reported PEC to collect multiple reducing equivalents at a central metal site while staying intact. Intramolecular electron transfer from the Ru(II)-based LAs to the Rh(III)-based EC subunit produces a doubly-reduced Rh metal center with the potential to deliver electrons to a substrate. Further modification of the [{(TL)_2_Ru(dpp)}_2_RhX_2_]^5+^ molecular components through halide variation, as well as TL variation, has generated a series of complexes functioning as PECs. In the presence of a sacrificial ED and H_2_O, many of the Rh centered PECs function as photocatalysts reducing H_2_O to H_2_[12,13,14,15,16]. Figure 1 displays an example of an ED-LA-BL-EC-BL-LA-ED structural motif for PEC and the required orbital energetics. Photoexcitation at 470 nm produces 7.2 ± 0.7 µmol of H_2_ in an CH_3_CN solvent system with 65 µM [{(bpy)_2_Ru(dpp)}_2_RhCl_2_]^5+^, 1.5 M DMA electron donor, 0.62 M H_2_O and 0.11 mM [CF_3_SO_3_^−^][DMAH^+^] after 5 h (DMA = *N,N*-dimethylaniline). Halide variation to the weaker σ-donating Br finely tunes the orbital energetics at the Rh site, while TL variation to phen or Ph_2_phen tunes the light absorbing capabilities of these systems with both expected to affect photocatalysis (Ph_2_phen = 4,7-diphenyl-1,10-phenanthroline). Systematic component variation within this series of Ru(II),Rh(III),Ru(II) trimetallics allows for careful analysis of the excited state properties that influence photocatalysis of H_2_O to H_2_.

Intermolecular electron transfer reactions have been widely studied focusing on the development of molecular photovoltaics [17,18,19,20]. Ru(II)-based polyazine LAs are efficient light absorbers throughout the UV and visible regions as photoexcitation populates ^3^MLCT (metal-to-ligand charge transfer) excited states with near unit efficiency that are photo- and redox-active. The prototypical light absorber [Ru(bpy)_3_]^2+^ undergoes optical excitation to populate an emissive ^3^MLCT electronic excited state that is both a more powerful oxidizing and reducing agent than the ground state species. Upon photoexcitation, this class of Ru(II)-polyazine LAs are known to undergo excited state oxidative and reductive quenching, Equations (1–3).

(1) $${\left[\text{Ru}{\left(\text{bpy}\right)}_{\text{3}}\right]}^{\text{2}+}\;\overset{h\nu }{\to }\;*{\left[\text{Ru}{\left(\text{bpy}\right)}_{\text{3}}\right]}^{\text{2}+}$$

*[Ru^II^(bpy)_3_]^2+^ + EA → [Ru^III^(bpy)_3_]^3+^ + EA^−^ (2)

*[Ru^II^(bpy)_3_]^2+^ + ED → [Ru^II^(bpy)_2_(bpy^−^)]^+^ + ED^+^ (3)

The rate of excited state electron transfer depends on the thermodynamic driving force for these reactions [4,21,22]. The excited state oxidation (Equation 4) and reduction (Equation 5) potentials of the excited LA are calculated using the energy of the E^0-0^ transition of the ^3^MLCT emission and the ground state redox potentials.

*E*(*LA^+^/LA) ≈ *E*(LA/LA^+^) − E^0-0^ (4)

*E*(*LA/LA^−^) ≈ *E*(LA/LA^−^) + E^0-0^ (5)

In the equations above, LA is the Ru(II)-polyazine light absorber, *E*(LA/LA^+^) is the ground state oxidation potential, *E*(LA/LA^−^) is the ground state reduction potential, *E*(*LA^+^/LA) is the excited state oxidation potential, and *E*(*LA/LA^−^) is the excited state reduction potential. Emission spectroscopy is often used to probe the rate of quenching of the emissive ^3^MLCT excited states by a quenching species, such as an ED [23,24]. Supramolecular complexes take advantage of covalently coupled molecular components to promote photoinduced intramolecular electron transfer. Bridging a Ru(II)-based LA to an EA subunit (LA-EA) can afford excited state intramolecular electron transfer upon photoexcitation of the LA subunit, as shown in Equations 6 and 7.

(6) $$\text{LA}-\text{EA}\overset{hv}{\to }*\text{LA}-\text{EA}$$

*LA-EA → LA^+^-EA^−^ (7)

Several Ru(II),Rh(III) bimetallic [25,26,27,28,29,30,31] and Ru(II),Rh(III),Ru(II) trimetallic [11,12,13,16,32] systems have been reported that undergo photoinduced intramolecular electron transfer. The Ru(II),Rh(III) bimetallic complexes [(bpy)_2_Ru(Mebpy-CH_2_CH(OH)CH_2_-Mebpy)Rh(bpy)_2_]^5+^ and [(Me_2_phen)_2_Ru(Mebpy-CH_2_CH_2_-Mebpy)Rh(Me_2_bpy)_2_]^5+^ (Mebpy = 4-methyl-2,2′-bipyridine; Me_2_phen = 4,7-dimethyl-1,10-phenanthroline; Me_2_bpy = 4,4’-dimethyl-2,2′-bipyridine) covalently couple the Ru-based LA and Rh-based EA through aliphatic BLs, affording rates of intramolecular electron transfer (k_et_) of 1.4 × 10^7^ and 1.7 × 10^8^ s^−1^, respectively [25,29]. Electronic communication between the two metal centers was negligible due to the nature of the BL and the distance between the metal centers. Modification of the BL to incorporate aromatic linkers displayed a strong dependence on the calculated k_et_ value to the distance between metal centers. With each additional phenylene linker in the Ru(II),Rh(III) complexes [(Me_2_phen)_2_Ru-bpy-(ph)_n_-bpy-Rh(Me_2_bpy)_2_]^5+^ (where n = 1, 2, or 3), values for k_et_ decrease by an order of magnitude with k_et_ = 3.0 × 10^9^ s^−1^, 4.3 × 10^8^ s^−1^ and 1.0 × 10^7^ s^−1^, respectively [26]. Similar to the aliphatic bridged Ru(II),Rh(III) bimetallics, the phenylene linker-containing complexes display negligible electronic communication between the metal centers. Further modification of the aromatic BL scaffold to include pyrazine components strongly influences k_et_. The complexes [(bpy)_2_Ru(dpp)Rh(bpy)_2_]^5+^, [(tpy)Ru(tpp)RhCl_3_]^2+^, and [(bpy)_2_Ru(dpp)RhCl_2_(phen)]^3+^ (tpy = 2,2′:6′,2′′-terpyridine; tpp = 2,3,5,6-tetrakis(2-pyridyl)pyrazine) have values of k_et_ = 2.8, 4.0, and 2.5 × 10^7^ s^−1^, respectively [30,31,33]. Substantial electronic communication between the metal centers greatly perturbs the observed photophysical properties of these coupled Ru(II),Rh(III) bimetallics.

**Figure 2:**
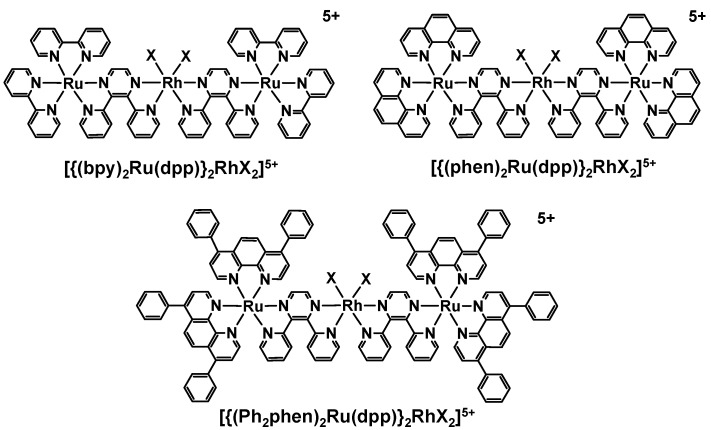
Ru(II),Rh(III),Ru(II) photoinitiated electron collectors of the supramolecular architecture [{(TL)_2_Ru(dpp)}_2_RhX_2_]^5+^ (TL = bpy = 2,2′-bipyridine, phen = 1,10-phenanthroline, Ph_2_phen = 4,7-diphenyl-1,10-phenanthroline; dpp = 2,3-bis(2-pyridyl)pyrazine; X = Cl or Br).

Reported herein is a study of the excited state dynamics and a kinetic analysis of the quenching of the ^3^MLCT excited states by the electron donor DMA as well as the kinetics of formation of a reduced Rh species within the supramolecular architecture [{(TL)_2_Ru(dpp)}_2_RhX_2_](PF_6_)_5_ (TL = bpy, phen or Ph_2_phen; X = Cl or Br), Figure 2. These supramolecules are known to function as photoinitiated electron collectors and photocatalysts in the reduction of H_2_O to H_2_. This structural motif provides for systems that are strong oxidizers in their excited state which allows use of electron donors and oxidation chemistry not accessible to typical [Ru(bpy)_3_]^2+^ based systems. The role of component modification on excited state dynamics and reaction rates is analyzed.

## 2. Results and Discussion

### 2.1. Photophysical Properties

**Figure 3:**
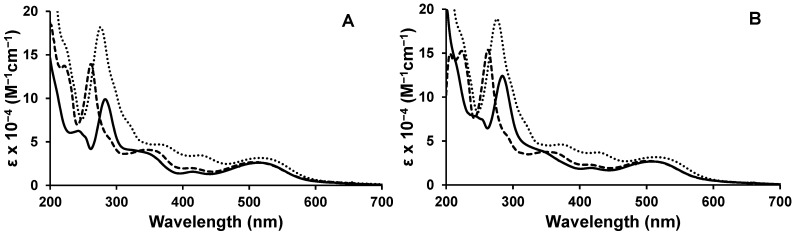
Electronic absorption spectra for the complexes (**A**) [{(TL)_2_Ru(dpp)}_2_RhCl_2_]^5+^, where TL = bpy (──), phen (- - -), Ph_2_phen (**· · ·**) and (**B**) [{(TL)_2_Ru(dpp)}_2_RhBr_2_]^5+^, where TL = bpy (──), phen (- - -), Ph_2_phen (**· · ·**).

The [{(TL)_2_Ru(dpp)}_2_RhX_2_]^5+^ trimetallic complexes are efficient light absorbers throughout the UV and visible regions at room temperature in acetonitrile, Figure 3. The UV region is dominated by intense TL π→π* intraligand (IL) transitions, with the dpp BL π→π* IL transitions occurring at slightly lower energy. The visible region displays higher energy Ru(dπ)→TL(π*) CT transitions and lowest energy Ru(dπ)→dpp(π*) CT transitions. The lowest-lying MLCT transition is nearly isoenergetic in the series of complexes indicative of the similar Ru(dπ)→dpp(π*) CT nature of the optically populated state. These systems absorb more of the solar spectrum than typical [Ru(bpy)_3_]^2+^ based systems via enhanced molar absorptivity in the UV and visible with Ru(dπ)→dpp(π*) CT transitions that provide absorption in the low energy visible.

**Figure 4:**
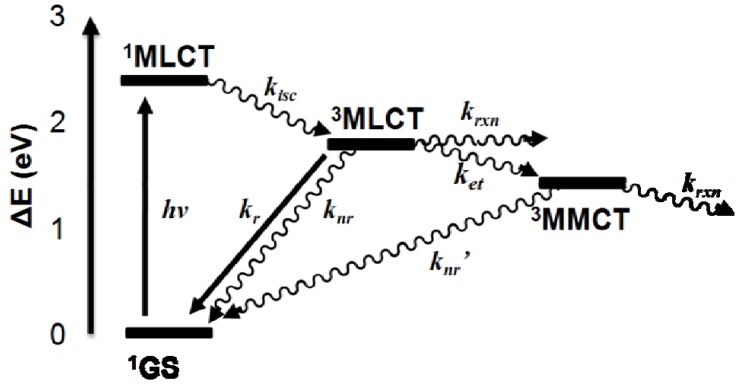
State diagram for [{(TL)_2_Ru(dpp)}_2_RhX_2_](PF_6_)_5_ trimetallic complexes, illustrated for [{(Ph_2_phen)_2_Ru(dpp)}_2_RhBr_2_](PF_6_)_5_ (TL = bpy, phen or Ph_2_phen; X = Cl or Br). *hν* is energy of the photon, *k_isc_* is the rate constant for intersystem crossing, *k_r_* is the rate constant for radiative decay, *k_nr_* is the rate constant for non-radiative decay, *k_et_* is the rate constant for intramolecular electron transfer, and *k_rxn_* is the rate constant for a photochemical reaction.

The excited state properties of the Ru(II),Rh(III),Ru(II) trimetallic and model Ru(II),Ru(II) bimetallic complexes are summarized in Table 1. The trimetallic complexes of the design [{(TL)_2_Ru(dpp)}_2_RhX_2_]^5+^ (TL = bpy, phen, Ph_2_phen; X = Cl or Br) display weak emission and a short excited state lifetime of the Ru(dπ)→dpp(π*) ^3^MLCT emissive excited state when compared with the model [(TL)_2_Ru(dpp)Ru(TL)_2_]^4+^ bimetallic complexes, which display the same Ru→μ-dpp ^3^MLCT emissive state but lack a Rh-based EC metal center. The Ru(II),Ru(II) bimetallic complexes are used as model systems for photophysical studies due to the similar nature and energy of the emissive Ru→μ-dpp CT excited state. Terminal ligand variation has been shown to modulate this ^3^MLCT emissive excited state (presumably from a contribution to the formally Ru(dπ) HOMO in this motif), therefore different Ru(II),Ru(II) bimetallics are needed for TL = bpy, phen, or Ph_2_phen [32]. Figure 4 displays the state diagram for the trimetallic complex [{(Ph_2_phen)_2_Ru(dpp)}_2_RhBr_2_](PF_6_)_5_. At RT, deactivation from the ^3^MLCT excited state is dominated by intramolecular electron transfer to populate a low-lying, energetically close Ru(dπ)→Rh(dσ*) ^3^MMCT excited state. This is supported by the observation of a Rh-based lowest unoccupied molecular orbital (LUMO) in electrochemical analyses of these systems [11,12,13,15,16]. The shortened excited state lifetime of the emissive ^3^MLCT state in the Ru(II),Rh(III),Ru(II) motif at RT is ascribed to intramolecular electron transfer to populate a low-lying ^3^MMCT state which quenches the ^3^MLCT state at RT but not at 77 K [32]. Due to the similar energy and nature of the emissive ^3^MLCT excited state for the Ru(II),Ru(II) bimetallic and Ru(II),Rh(III),Ru(II) trimetallic complexes, it is assumed that calculated rate constants for radiative (k_r_) and non-radiative (k_nr_) decay from the ^3^MLCT excited state of the bimetallics are the same for the analogous trimetallics. Both the title trimetallics and the model bimetallic used as the model for each trimetallic possess not only the same Ru(dπ)→dpp(π*) ^3^MLCT emissive state but also the same TL and the same (TL)_2_Ru^II^(∝-dpp) sub-unit. Under this assumption, the rate constant for intramolecular electron transfer (k_et_) to populate the non-emissive ^3^MMCT excited state was calculated and varies from (1.4–2.8) × 10^7^ s^−1^ in this series of complexes. Within each series of TL = bpy, phen, or Ph_2_phen trimetallics, varying the halide from Cl to Br displays a decrease in Φ^em^ and τ with a subsequent increase in k_et_. The inclusion of the weaker σ-donating Br stabilizes the ^3^MMCT excited state and affords enhanced driving force and rate of intramolecular electron transfer to populate the ^3^MMCT state. Choice of TL within the Ru(II),Rh(III),Ru(II) architecture also impacts the excited state properties with the energy, Φ^em^, and τ of the formally Ru(dπ)→dpp(π*) ^3^MLCT excited state varying. The phen systems display enhanced rates of intramolecular electron transfer to populate the ^3^MMCT state *vs.* bpy or Ph_2_phen. The phen systems have slightly higher energy ^3^MLCT excited states which may provide a larger driving force for electron transfer to populate the ^3^MMCT state.

**Table 1:** Photophysical Properties of [{(TL)_2_Ru(dpp)}_2_RhX_2_]^5+^ Trimetallic and Analogous [(TL)_2_Ru(dpp)Ru(TL)_2_]^4+^ Bimetallic Complexes at Room Temperature and 77 K.

Complex	RT *^a^*						77 K *^b^*	
	λ_max_^em^ (nm)	Φ_MLCT_^em^ (10^−3^) *^c^*	τ (ns) *^d^*	k_r_ (10^3^ s^−1^)	k_nr_ (10^6^ s^−1^)	k_et_ (10^7^ s^−1^)	λ_max_^em^ (nm)	τ (μs) *^d^*
[(bpy)_2_Ru(dpp)Ru(bpy)_2_]^4+^	752	0.97	145	6.7	6.9		730	2.4
[(phen)_2_Ru(dpp)Ru(phen)_2_]^4+^	750	1.6	170	9.4	5.9		695	2.0
[(Ph_2_phen)_2_Ru(dpp)Ru(Ph_2_phen)_2_]^4+^	754	1.7	192	9.0	5.2		698	2.0
[{(bpy)_2_Ru(dpp)}_2_RhCl_2_]^5+^	776	0.26	38	6.7	6.9	1.9	716	1.9
[{(bpy)_2_Ru(dpp)}_2_RhBr_2_]^5+^	776	0.14	34	6.7	6.9	2.3	716	1.9
[{(phen)_2_Ru(dpp)}_2_RhCl_2_]^5+^	760	0.22	35	9.4	5.9	2.3	706	1.8
[{(phen)_2_Ru(dpp)}_2_RhBr_2_]^5+^	760	0.17	30	9.4	5.9	2.8	706	1.9
[{(Ph_2_phen)_2_Ru(dpp)}_2_RhCl_2_]^5+^	770	0.24	52	9.0	5.2	1.4	696	1.8
[{(Ph_2_phen)_2_Ru(dpp)}_2_RhBr_2_]^5+^	770	0.20	40	9.0	5.2	2.0	696	1.9

*^a^* Measured in acetonitrile at room temperature; *^b^* Measured in 4:1 EtOH/MeOH rigid matrix at 77 K; *^c^* Reported values of Φ ± 5 %; *^d^* Reported values of τ ± 5 %.

At 77 K in a rigid glass matrix, the Ru(II),Rh(III),Ru(II) trimetallic and Ru(II),Ru(II) bimetallic complexes display similar emissive excited states with nearly equivalent lifetimes. The shape of the ^3^MLCT emission profile sharpens in rigid media at 77 K and the emission maxima blue shift. This is consistent with electron transfer at RT to populate the ^3^MMCT state from the emissive ^3^MLCT state being impeded at 77 K in a rigid media.

### 2.2. Photochemical Properties

**Figure 5:**
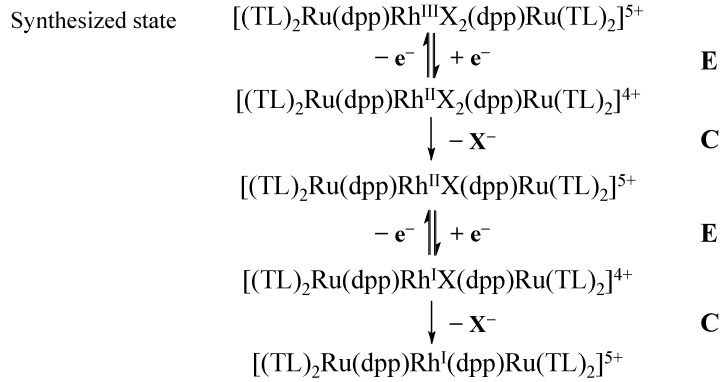
Mechanism for the electrochemical reduction of [{(TL)_2_Ru(dpp)}_2_RhX_2_]^5+^ (TL = bpy, phen or Ph_2_phen; X = Cl or Br).

Photochemical reduction of these [{(TL)_2_Ru(dpp)}_2_Rh^III^X_2_]^5+^ trimetallic complexes illustrates their ability to undergo photoinitiated electron collection at the Rh(III) metal center to generate Rh(I) centered trimetallics. When illuminated at 470 nm in the presence of the sacrificial electron donor DMA, the electronic absorption spectrum displays a shift to higher energy of the Ru(dπ)→dpp(π*) CT transition due to the formation of a more electron-rich Rh(I) metal center. The increase in electron density at Rh destabilizes the dpp(π*) acceptor orbitals relative to Rh(III) resulting in an increase in the energy of the observed Ru(dπ)→dpp(π*) CT transition. The electronic absorption spectra generated through electrochemical reduction of the Rh(III) to Rh(I) metal center correlates well with the photochemical reduction of trimetallics [11]. The reduction of these complexes occurs via an ECEC mechanism analogous to previously studied [Rh(NN)_2_X_2_]^+^ , Figure 5 [34,35].

The photochemical reduction of the complexes [{(TL)_2_Ru(dpp)}_2_Rh^III^X_2_]^5+^ is critical to photocatalysis and involves many steps including possible reactions from the ^3^MLCT and ^3^MMCT excited states. Generation of the two electron reduced species [{(TL)_2_Ru(dpp)}_2_Rh^I^]^5+^ forms through a Rh(II) intermediate as observed electrochemically with [Rh(bpy)_2_Cl_2_]^+^ and [Rh(dpp)_2_Br_2_]^+^ [34,35]. Photochemically, this reduced species can be formed through intermolecular electron transfer from a sacrificial electron donor to the ^3^MLCT or ^3^MMCT excited states. Additionally, both excited states can undergo unimolecular or bimolecular deactivation. The present kinetic study will analyze the rate of intramolecular electron transfer described above, the rate of quenching of the ^3^MLCT state via Stern-Volmer analysis and the rate of photochemical reduction via spectroscopic analysis. These pathways are all critical to formation of the Ru(II),Rh(II),Ru(II) form of the complexes. Equations 8–15 display the various kinetic pathways for the formation of the singly-reduced Ru(II),Rh(II),Ru(II) photoreduced product. The [{(TL)_2_Ru(dpp)}_2_RhX_2_]^5+^ trimetallic complexes are represented as Ru^II^(dpp)Rh^III^(dpp)Ru^II^.

MLCT Excitation:
  (8) $${\text{Ru}}^{\text{II}}\left(\text{dpp}\right){\text{Rh}}^{\text{III}}\left(\text{dpp}\right){\text{Ru}}^{\text{II}}\overset{h\nu }{\to }*{\text{Ru}}^{\text{III}}({\text{dpp}}^{-}){\text{Rh}}^{\text{III}}\left(\text{dpp}\right){\text{Ru}}^{\text{II}}$$
Unimolecular Deactivation of ^3^MLCT State:
  (9) $$*{\text{Ru}}^{\text{III}}({\text{dpp}}^{-}){\text{Rh}}^{\text{III}}\left(\text{dpp}\right){\text{Ru}}^{\text{II}}\overset{k_{1}}{\to }{\text{Ru}}^{\text{II}}\left(\text{dpp}\right){\text{Rh}}^{\text{III}}\left(\text{dpp}\right){\text{Ru}}^{\text{II}}$$
Bimolecular Deactivation of ^3^MLCT State:
  (10) $$\text{DMA }+\;*{\text{Ru}}^{\text{III}}({\text{dpp}}^{-}){\text{Rh}}^{\text{III}}\left(\text{dpp}\right){\text{Ru}}^{\text{II}}\overset{k_{2}}{\to }{\text{Ru}}^{\text{II}}\left(\text{dpp}\right){\text{Rh}}^{\text{III}}\left(\text{dpp}\right){\text{Ru}}^{\text{II}}+\text{ DMA}$$
Reductive Quenching of ^3^MLCT State:
  (11) $$\text{DMA }+\;*{\text{Ru}}^{\text{III}}({\text{dpp}}^{-}){\text{Rh}}^{\text{III}}\left(\text{dpp}\right){\text{Ru}}^{\text{II}}\overset{k_{q}}{\to }{\text{Ru}}^{\text{II}}\left(\text{dpp}\right){\text{Rh}}^{\text{II}}\left(\text{dpp}\right){\text{Ru}}^{\text{II}}+{\text{ DMA}}^{+}$$
Intramolecular Electron Transfer:
  (12) $$*{\text{Ru}}^{\text{III}}({\text{dpp}}^{-}){\text{Rh}}^{\text{III}}\left(\text{dpp}\right){\text{Ru}}^{\text{II}}\overset{k_{et}}{\to }*{\text{Ru}}^{\text{III}}\left(\text{dpp}\right){\text{Rh}}^{\text{II}}\left(\text{dpp}\right){\text{Ru}}^{\text{II}}$$
Unimolecular Deactivation of ^3^MMCT State:
  (13) $$*{\text{Ru}}^{\text{III}}({\text{dpp}}^{-}){\text{Rh}}^{\text{III}}\left(\text{dpp}\right){\text{Ru}}^{\text{II}}\overset{k_{4}}{\to }*{\text{Ru}}^{\text{III}}\left(\text{dpp}\right){\text{Rh}}^{\text{II}}\left(\text{dpp}\right){\text{Ru}}^{\text{II}}$$
Bimolecular Deactivation of ^3^MMCT State:
  (14) $$\text{DMA }+\;*{\text{Ru}}^{\text{III}}\left(\text{dpp}\right){\text{Rh}}^{\text{II}}\left(\text{dpp}\right){\text{Ru}}^{\text{II}}\overset{k_{3}}{\to }{\text{Ru}}^{\text{II}}\left(\text{dpp}\right){\text{Rh}}^{\text{III}}\left(\text{dpp}\right){\text{Ru}}^{\text{II}}+\text{ DMA}$$
Reductive Quenching of ^3^MLCT State:
  (15) $$\text{DMA }+\;*{\text{Ru}}^{\text{III}}\left(\text{dpp}\right){\text{Rh}}^{\text{II}}\left(\text{dpp}\right){\text{Ru}}^{\text{II}}\overset{k_{q2}}{\to }{\text{Ru}}^{\text{II}}\left(\text{dpp}\right){\text{Rh}}^{\text{II}}\left(\text{dpp}\right){\text{Ru}}^{\text{II}}+{\text{ DMA}}^{+}$$

Using our mechanism, unimolecular deactivations k_1_ and k_4_ include radiative, k_r_, and non-radiative, k_nr_, decay including relaxation mediated by solvent. Bimolecular deactivations, k_2_ and k_3_, include electron transfer from DMA followed by rapid back electron transfer as well as other bimolecular deactivations by DMA.

### 2.3. Emission Quenching

The emissive nature of the ^3^MLCT excited state provides a handle to study the excited state dynamics. This probe was used to study the rate of intramolecular electron transfer (k_et_) as described above. Addition of the ED DMA provides a means to assay the kinetics of quenching of the ^3^MLCT state by this ED. The sacrificial electron donor DMA has been shown to quench the ^3^MLCT emissive excited state of Ru-polyazine complexes and [{(bpy)_2_Ru(dpp)}_2_RhCl_2_]^5+^ through bimolecular interactions [11]. The [{(TL)_2_Ru(dpp)}_2_RhX_2_]^5+^ trimetallic complexes reported herein undergo efficient excited state reductive quenching of the ^3^MLCT emission. DMA is reported to quench the ^3^MLCT emission of [Ru(bpy)_3_]^2+^ [23,36] and [Ru(bpz)_3_]^2+^ (bpz = 2,2′-bipyrazine) [37] with a rate constant of 7.1 × 10^7^ M^−1^s^−1^ and 8.4 × 10^9^ M^−1^s^−1^, respectively. The ^3^MLCT excited state of *[Ru(bpy)_3_]^2+^ and *[Ru(bpz)_3_]^2+^ have excited state reduction potentials of 0.82 V and 1.50 V *vs.* Ag/AgCl, respectively, while DMA has a ground state oxidation potential of 0.86 V *vs.* Ag/AgCl [36]. The thermodynamic driving force (*E*_redox_) for reductive quenching of the ^3^MLCT excited state is determined by the ground state oxidation potential of the electron donor (*E*(ED^0/+^)) and the excited state reduction potential of the Ru(II)-polyazine complex (*E*(*CAT^n+^/CAT^(n − 1)+^)), Equations 16 and 17:

E_redox_ = E(*CAT^n+^/CAT^(n − 1)+^) − E(ED^0/+^) (16)

E(*CAT^n+^/CAT^(n − 1)+^) = E^0-0^ + E(CAT^n+^/CAT^(n − 1)+^) (17)

where *E*(CAT^n+^/CAT^(n − 1)+^) is the ground state reduction potential of the complex and E^0-0^ is the energy of the 0-0 transition between the excited state and the ground state. The E^0-0^ energy is estimated using the observed 77 K emission maxima. Using the above calculations, *E*_redox_ for the excited state reductive quenching of *[Ru(bpy)_3_]^2+^ by DMA is a thermodynamically unfavorable process (*E*_redox_ = −0.04 V) and supports the observed low rate, 7.1 × 10^7^ M^−1^s^−1^. Conversely, *[Ru(bpz)_3_]^2+^ has a thermodynamically favorable value of *E*_redox_ (+0.64 V) for excited state reductive quenching using DMA with a larger rate, 8.4 × 10^9^ M^−1^s^−1^, close to the diffusion control limit [38]. The thermodynamic driving force strongly impacts the excited state reductive quenching of these Ru(II)-polyazine complexes [23,37,39]. [{(TL)_2_Ru(dpp)}_2_RhX_2_]^5+^ trimetallic complexes display positive values of *E*_redox_ for the reductive quenching of the ^3^MLCT, as well as ^3^MMCT, excited states with the ^3^MLCT quenching being similar to the *[Ru(bpz)_3_]^2 +^ ^3^MLCT excited state, Table 2. The title trimetallics are strong oxidizers in their ^3^MLCT excited state with *E*(*CAT^n+^/CAT^(n − 1)+^) ranging from 1.35–1.46 V *vs.* Ag/AgCl providing the driving force to oxidize many molecules including some water oxidation catalysts.

**Table 2:** Excited state reduction potentials and thermodynamic driving force for excited state reductive quenching of [{(TL)_2_Ru(dpp)}_2_RhX_2_]^5+^ supramolecular complexes.

Complex	*E*(*CAT^n+^/CAT^(n − 1)+^) ^3^MLCT (V)*^a^*	*E*(*CAT^n+^/CAT^(n − 1)+^) ^3^MMCT (V)*^a^*	*E*_redox_^3^MLCT (V)*^b^*	*E*_redox_^3^MMCT (V)*^b^*	k_q_ + k_2_ (M^−1^s^−1^)*^c^*
[Ru(bpy)_3_]^2+^ *^e^*	+0.82	--	−0.04	--	7.1 × 10^7^ *^d^*
[Ru(bpz)_3_]^2+^ *^f^*	+1.50	--	+0.64	--	8.4 × 10^9^ *^d^*
[{(bpy)_2_Ru(dpp)}_2_RhCl_2_]^5+^	+1.35	+0.94	+0.49	+0.08	2.5 × 10^9^
[{(bpy)_2_Ru(dpp)}_2_RhBr_2_]^5+^	+1.38	+0.99	+0.52	+0.13	3.2 × 10^9^
[{(phen)_2_Ru(dpp)}_2_RhCl_2_]^5+^	+1.41	+1.01	+0.55	+0.15	3.9 × 10^9^
[{(phen)_2_Ru(dpp)}_2_RhBr_2_]^5+^	+1.44	+1.05	+0.58	+0.19	5.9 × 10^9^
[{(Ph_2_phen)_2_Ru(dpp)}_2_RhCl_2_]^5+^	+1.43	+1.04	+0.57	+0.18	1.5 × 10^9^
[{(Ph_2_phen)_2_Ru(dpp)}_2_RhBr_2_]^5+^	+1.46	+1.09	+0.60	+0.23	2.9 × 10^9^

*^a^* Potential in V *vs.* Ag/AgCl, *E*(*CAT^n+^/CAT^(n − 1)+^) is the excited state reduction potential; *^b^* Thermodynamic driving force calculated by measuring the difference between the excited state reduction potential of the complex and the ground state oxidation potential of the electron donor DMA (DMA^0/+^ = 0.86 V *vs.* Ag/AgCl); ^c^ Rate constant for quenching of ^3^MLCT excited state through bimolecular interactions with the electron donor DMA; ^d^ Values are reported k_q_ rate constants; ^e^ From reference [33]; ^f^ From reference [34].

**Figure 6:**
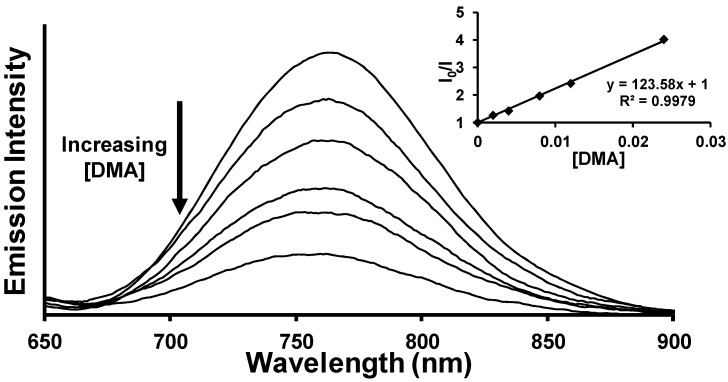
Emission quenching of [{(phen)_2_Ru(dpp)}_2_RhCl_2_]^5+^ ^3^MLCT excited state using DMA sacrificial electron donor. Inset: Stern-Volmer plot depicting linear relationship of emission quenching and DMA concentration.

A Stern-Volmer analysis was performed to observe the ^3^MLCT emission quenching of the trimetallic complexes using the electron donor DMA, Figure 6. All complexes show a linear Stern-Volmer relationship with reduction of the ^3^MLCT excited state emission intensity varying linearly with increasing [DMA]. Equation 18 relates the ratio of the intensity of ^3^MLCT emission in the absence (I_0_) and presence (I) of DMA to the concentration of DMA added:

(18) $$\frac{{\text{I}}_{0}}{\text{I}}=\frac{\left({\text{k}}_{\text{q}}+\;{\text{k}}_{2})[\mathrm{DMA}\right]}{\left({\text{k}}_{1}+\;{\text{k}}_{\text{et}}\right)}+\;1$$

where k_1_ = k_r_ + k_nr_ [38]. The slope of the Stern-Volmer quenching plot contains the rate constant for quenching by DMA of the ^3^MLCT state via bimolecular deactivation (k_2_) or photoreduction (k_q_) to form the reduced Rh(II) photoproduct. From these experiments, the values corresponding to the deactivation of the ^3^MLCT excited state through bimolecular interactions with DMA (k_q_ + k_2_) were obtained and vary from 1.5 × 10^9^ M^−1^s^−1^ to 5.9 × 10^9^ M^−1^s^−1^, indicative of efficient quenching of the ^3^MLCT excited state, Table 2. Competing pathways for deactivation of the ^3^MLCT excited state are radiative (k_r_) and non-radiative (k_nr_) decay to the ^1^GS and intramolecular electron transfer (k_et_) to populate the ^3^MMCT excited state. The calculated rate constants for these unimolecular deactivation pathways (Table 1) are substantially smaller than the DMA bimolecular quenching rate constants. This observation suggests that in the presence of DMA, the dominating pathways of deactivation from the ^3^MLCT excited state involve bimolecular quenching with the electron donor. While this Stern-Volmer analysis of the ^3^MLCT excited state quenching does not permit the independent calculation of k_q_ and k_2_, photochemical product formation studies enable extraction of the k_q_ value and the subsequent value of k_2_ is obtained below.

### 2.4. Product Formation

**Figure 7:**
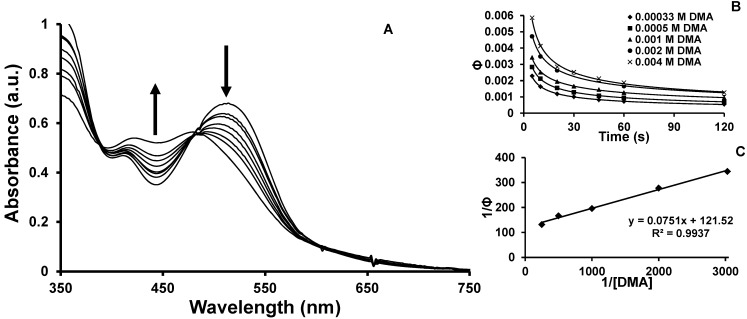
**(A)** Photochemical reduction of [{(phen)_2_Ru(dpp)}_2_RhCl_2_]^5+^ using DMA sacrificial electron donor to generate reduced Rh photoproduct; (**B**) Analysis showing non-linear relationship of product formation with respect to photolysis time at varying DMA concentrations; (**C**) Stern-Volmer plot depicting linear relationship between reciprocal of product formation and reciprocal of DMA concentration.

The photochemical reduction of the [{(TL)_2_Ru(dpp)}_2_RhX_2_]^5+^ complexes by two electrons to produce Rh(I) species is expected to proceed through a Rh(II) intermediate. The spectroscopic change upon reduction from Rh(III) to Rh(II) to Rh(I) proceeds with a smooth shift to higher energy of the Ru(dπ)→dpp(π*) CT transition. This photoproduct can be generated through excited state reductive quenching of the ^3^MLCT or ^3^MMCT excited states, or a combination of both states as depicted in Equations 11 and 15. Monitoring changes to the electronic absorption spectra over time while photolyzing the [{(TL)_2_Ru(dpp)}_2_RhX_2_]^5+^ complexes in the presence of varied concentrations of DMA provides a means of analyzing the rate constants for photoreduction of the Rh center. The Stern-Volmer kinetic relationship for excited state reactivity in this forum is applied to analyze the kinetics of the ^3^MLCT or ^3^MMCT bimolecular photoreduction using Equations 19 and 20. A Stern-Volmer analysis of the quantum yield for product formation was undertaken to assist in exploring the role that ^3^MLCT and ^3^MMCT excited states have on supramolecule reduction. Upon photolysis with visible light (λ = 470 nm), the lowest energy Ru(dπ)→dpp(π*) CT transition *ca.* 518 nm decreases intensity with a concurrent shift to higher energy *ca.* 440 nm. The change in absorbance of these two transitions at 518 and 440 nm are monitored as a function of time and the data extrapolated to t = 0 for analysis, Figure 7A. Figure 7B and Figure 7C correspond to the overall quantum yield of reduced Rh product formation.

Product formation to generate the reduced supramolecules can occur from the ^3^MLCT or ^3^MMCT excited states. Kinetic analysis first considers product formation from the ^3^MLCT state. The Ru(dπ)→dpp(π*) ^3^MLCT excited state can deactivate through unimolecular deactivation to the ground state (k_1_), bimolecular deactivation through interaction with DMA (k_2_), intramolecular electron transfer to populate the ^3^MMCT state (k_et_), or reductive quenching by DMA to produce the reduced species (k_q_). Equation 19 relates the quantum yield of formation of the reduced species (Φ_product_) to [DMA].

(19) $$\frac{1}{{\text{Φ}}_{\text{product}}}=\frac{{\text{k}}_{1}+{\text{ k}}_{\text{et}}}{{\text{k}}_{\text{q}}[\text{DMA}]}+\frac{{\text{k}}_{\text{q}}+{\text{ k}}_{2}}{{\text{k}}_{\text{q}}}$$

Plotting 1/Φ_product_ *vs.* 1/[DMA] gives a linear relationship, with a slope of (k_1_+k_et_)/k_q_ and an intercept of (k_q_+k_2_)/k_q_. The rate constant for unimolecular deactivation, k_1_, is the sum of k_r_ and k_nr_ and has been determined above. The rate constant for intramolecular electron transfer, k_et_, was obtained from our above emission analysis. This allows the determination of k_q_ and k_2_, Table 3.

**Table 3:** Rate constants for Rh reduction for [{(TL)_2_Ru(dpp)}_2_RhX_2_](PF_6_)_5_ supramolecular complexes from the ^3^MLCT and ^3^MMCT excited states.

Complex	k_q_ (10^8^ M^−1^s^−1^) *^a^*	k_2_ (10^9^ M^−1^s^−1^) *^b^*	Φ_3MMCT_ *^c^*	k_4_/k_q2_ *^d^*
[{(bpy)_2_Ru(dpp)}_2_RhCl_2_]^5+^	2.1	2.3	0.74	0.094
[{(bpy)_2_Ru(dpp)}_2_RhBr_2_]^5+^	3.5	2.9	0.77	0.066
[{(phen)_2_Ru(dpp)}_2_RhCl_2_]^5+^	3.8	3.5	0.79	0.060
[{(phen)_2_Ru(dpp)}_2_RhBr_2_]^5+^	4.3	5.5	0.82	0.063
[{(Ph_2_phen)_2_Ru(dpp)}_2_RhCl_2_]^5+^	2.5	1.3	0.73	0.056
[{(Ph_2_phen)_2_Ru(dpp)}_2_RhBr_2_]^5+^	4.2	2.4	0.79	0.047

*^a^* Rate constant for reductive quenching of the ^3^MLCT excited state with DMA; *^b^* Rate constant for bimolecular deactivation through interaction with DMA; *^c^* Quantum yield of formation of the ^3^MMCT excited state; *^d^* Ratio of the rate constant for back electron transfer from the ^3^MMCT excited state to the rate constant for reduction quenching from the ^3^MMCT state with DMA.

Reduction of the [{(TL)_2_Ru(dpp)}_2_RhX_2_]^5+^ can also occur from the ^3^MMCT excited state. The Ru(dπ)→Rh(σ*) ^3^MMCT excited state can undergo multiple deactivation pathways including unimolecular deactivation (k_4_), bimolecular deactivation with DMA (k_3_) and reductive quenching of the excited state by DMA to produce the singly reduced species (k_q2_). The efficiency of Rh(II) product formation from the ^3^MMCT state depends on the efficiency of populating the ^3^MMCT state (Φ_3MMCT_). Equation 20 relates the quantum yield of formation of the reduced species (Φ_product_) from the ^3^MMCT excited state to [DMA].

(20) $$\frac{1}{{\text{Φ}}_{\text{product}}}=\left(\frac{1}{{\text{Φ}}_{3_{\text{MMCT}}}}\right)\left(\frac{{\text{k}}_{4}}{{\text{k}}_{\text{q}2}\left[\text{DMA}\right]}\right)+\frac{{\text{k}}_{\text{q}2}+{\text{k}}_{3}}{{\text{k}}_{\text{q}2}}$$

A plot of 1/Φ_product_ and 1/[DMA] is linear with a slope of (1/Φ_3MMCT_)(k_4_/k_q2_) and an intercept of (k_q2_ + k_3_)/k_q2_. Values obtained for Φ_3MMCT_ and k_4_/k_q2_ from these analyses are presented in Table 3. The Φ_3MMCT_ is given by the ratio of k_et_ to k_r_+k_nr_ determined from the emission of the [{(TL)_2_Ru(dpp)}_2_RhX_2_]^5+^ complexes above. A direct measure of k_4_ is not provided so this analysis gives a ratio of k_4_/k_q2_. The Ru(II),Rh(III) bimetallic complex [(Me_2_phen)_2_Ru(Mebpy-CH_2_CH_2_-Mebpy) Rh(Me_2_bpy)_2_]^5+^ (Me_2_phen = 4,7-dimethyl-1,10-phenanthroline; Me_2_bpy = 4,4’-dimethyl-2,2′-bipyridine) was studied via transient spectroscopy to provide k_4_ = 7.1 × 10^9^ s^−1^ [25]. This system shows a k_et_ to populate the ^3^MMCT state of 1.4 × 10^7^ s^−1^, similar in magnitude to our systems. The rate of back electron transfer from Rh(II) to Ru(III) to generate the ground state from the ^3^MMCT state, k_4_, is expected to be fast for our complexes given the direct dpp coupling of the Ru and Rh centers in our systems *vs.* the Mebpy-CH_2_CH_2_-Mebpy linker in the previously reported system. Assuming k_4_ for our systems is >7.1 × 10^9^ s^−1^, this calculates k_q2_ values of *ca*. 10^11^ M^−1^s^−1^, an unreasonably large number. This suggests supramolecule reduction occurs primarily out of the ^3^MLCT state in our systems. The direct analysis of the contribution of the ^3^MMCT state to product formation is not accessible via these methods. The analysis herein does highlight that any photoreduction via the ^3^MMCT state would occur on the picosecond time scale.

Several pathways of deactivation of the ^3^MLCT state impact the trimetallic complexes’ ability to function as PECs and ultimately as solar energy conversion catalysts for water reduction. Deactivation of the ^3^MLCT state to the GS is a dominant pathway both via non-radiative (k_nr_), radiative (k_r_), and bimolecular deactivation (k_2_). The quenching of the ^3^MLCT excited state of the trimetallic complexes [{(TL)_2_Ru(dpp)}_2_RhX_2_]^5+^ in CH_3_CN at RT is very efficient with rate constants 1–6 × 10^9^ M^−1^s^−1^ at the diffusion control limit. The rate of the associated photoreduction of the trimetallics by DMA is less, 2–4 × 10^8^ M^−1^s^−1^, indicative of the often efficient back electron transfer prior to cage escape in Ru-polyazine systems. Nonetheless photoreduction occurs at a significant rate, 10^8^ M^−1^s^−1^, providing for the rapid conversion of the Rh(III) supramolecules to reduced species. The variation of the halide bound to the Rh from Cl to Br provides for enhanced rates of photoreduction independent of TL (bpy, phen or Ph_2_phen). TL variation impacts observed rates as well. Emission quenching by DMA (k_q_ + k_2_) is most efficient for phen complexes with Ph_2_phen providing for the lowest rate of DMA quenching of the ^3^MLCT excited state. The enhanced rate of quenching of the ^3^MLCT state by DMA for TL = phen may be a result of efficient π-π interaction of the phen TL with the DMA electron donor placing the DMA near the Ru center.

The above Ru(II),Rh(III),Ru(II) trimetallic complexes are photocatalysts in the reduction of H_2_O to H_2_, Table 4. The photosystems containing 65 μM photocatalyst, 1.5 M DMA, 0.62 M H_2_O and 0.11 mM [CF_3_SO_3_^−^][DMAH^+^] in CH_3_CN were photolyzed for 5 h using a 470 nm LED light source. Turnover numbers (TON) were measured as the mol of H_2_ produced per mol of Rh catalytic center. The quantum efficiency of H_2_ (Φ_H2_) was measured as mol of H_2_ produced per mol of photons, multiplied by two given the formation of H_2_ is a two photon and two electron process within our molecular architecture. Halide variation from Cl to Br displays more efficient H_2_ production as suggested by the enhanced rates of reduced Rh product formation. Photocatalysts where TL = phen display the lowest amount of H_2_, consistent with the larger rate constant for bimolecular deactivation of the ^3^MLCT excited state (k_2_) inhibiting efficient formation of the reduced Rh species. While photocatalysts with TL = Ph_2_phen outperform TL = bpy or phen systems, the observed excited state rate constants do not vary greatly, suggesting additional factors impact photocatalytic functioning. The steric demands of the Ph_2_phen ligand may provide protection of the photoreduced Rh(I) center, decreasing unfavorable side reactions and therefore enhancing H_2_ production.

**Table 4:** Photocatalytic H_2_ Production from Water using [{(TL)_2_Ru(dpp)}_2_RhX_2_](PF_6_)_5_ Supramolecular Complexes.

Complex *^a^*	H_2_ (μmol)	TON *^b^*	Φ_H2_ *^c^*
[{(bpy)_2_Ru(dpp)}_2_RhCl_2_]^5+^	7.2 ± 0.7	25 ± 2	0.0025
[{(bpy)_2_Ru(dpp)}_2_RhBr_2_]^5+^	8.9 ± 0.4	31 ± 1	0.0055
[{(phen)_2_Ru(dpp)}_2_RhCl_2_]^5+^	4.1 ± 0.2	14 ± 1	0.0017
[{(phen)_2_Ru(dpp)}_2_RhBr_2_]^5+^	5.9 ± 0.7	20 ± 3	0.0026
[{(Ph_2_phen)_2_Ru(dpp)}_2_RhCl_2_]^5+^	33 ± 3	110 ± 10	0.012
[{(Ph_2_phen)_2_Ru(dpp)}_2_RhBr_2_]^5+^	40 ± 4	140 ± 10	0.019

*^a^* Results correspond to 5 h photolysis time using 470 nm LED light source (light flux = 2.36 ± 0.05 × 10^19^ photons/min; solution volume = 4.5 mL; head space volume = 15.5 mL); *^b^* TON = turnover numbers = mol H_2_ produced per mol Rh catalytic center; *^c^* Φ_H2_ = maximum quantum efficiency of H_2_ formation.

## 3. Experimental Section

### 3.1. Materials

All solvents and chemicals were used as received unless otherwise stated. Spectral grade acetonitrile was purchased from Burdick and Jackson. Redistilled *N,N*-dimethylaniline was purchased from Aldrich Chemical Company. The complexes [{(bpy)_2_Ru(dpp)}_2_RhCl_2_](PF_6_)_5_ [11], [{(bpy)_2_Ru(dpp)}_2_RhBr_2_](PF_6_)_5_ [12], [{(phen)_2_Ru(dpp)}_2_RhCl_2_](PF_6_)_5_ [13], [{(phen)_2_Ru(dpp)}_2_RhBr_2_](PF_6_)_5_ [16], [{(Ph_2_phen)_2_Ru(dpp)}_2_RhCl_2_](PF_6_)_5_ [15] and [{(Ph_2_phen)_2_Ru(dpp)}_2_RhBr_2_](PF_6_)_5_ [15] were prepared as reported.

### 3.2. Methods

#### 3.2.1. Electronic Absorption Spectroscopy

Electronic absorption spectra were measured using a Hewlett-Packard 8452A diode array spectrophotometer with 2 nm resolution. Spectra were recorded at room temperature in spectral grade acetonitrile using a 1 cm path length cylindrical quartz cuvette (Starna Cells, Inc., Atascadero, CA, USA).

#### 3.2.2. Steady State Luminescence Spectroscopy

The room temperature steady state emission spectra were measured in spectral grade acetonitrile using a 1 cm path length quartz cuvette equipped with a screw top (Starna Cells, Inc.; Atascadero, CA, USA). The instrument used to record the spectra was a QuantaMaster Model QM-200-45E fluorimeter from Photon Technologies International, Inc. The excitation source was a water-cooled 150 W Xenon arc lamp with the corresponding emission collected at a 90° angle using a thermoelectrically cooled Hamamatsu 1527 photomultiplier tube operating in photon counting mode with 0.25 nm resolution. The emission monochromator contained a Czerny-Turner style grating monochromator set to 1,200 line/mm 750 nm blaze.

#### 3.2.3. Excited State Emission Quenching

Stock solutions of each trimetallic complex were prepared using spectral grade acetonitrile. Sample solutions were composed of a fixed final concentration of trimetallic complex (~30 μM) in a 1 cm quartz cuvette with increasing final concentrations of DMA ((2.4–0.2) × 10^−2^ M) added to a new sample. DMA was injected into the sample in the dark using a syringe just prior to excitation from the 150 W Xe arc lamp light source. The steady state emission spectrum for each sample was obtained and a Stern-Volmer plot of I_0_/I *vs.* [DMA] was generated and analyzed [38].

#### 3.2.4. Photochemical Product Formation

Sample solutions were composed of a fixed concentration of trimetallic complex (~25 μM) with increasing final concentrations of DMA ((4.0–0.33) × 10^−3^ M) added to each sample. The electronic absorption spectra were measured after photolysis on a 470 nm LED array designed and constructed locally (flux = 2.83 × 10^19^ photons/min) [40]. Data were plotted and extrapolated to zero time.

#### 3.2.5. Photocatalytic Hydrogen Production

The photocatalytic hydrogen production experiments were performed using modifications of previously reported conditions [14]. The trimetallic stock solutions (92 μM) in CH_3_CN were combined with water (acidified to pH 2 using CF_3_SO_3_H) in air tight photolysis reaction cells that were deoxygenated using argon gas. The electron donor DMA was deoxygenated separately and injected into the reaction cells just prior to photolysis (final conditions: [trimetallic] = 65 μM; [DMA] = 1.5 M; [H_2_O] = 0.62 M; [DMAH^+^][CF_3_SO_3_^−^] = 0.11 mM; solution volume = 4.5 mL; headspace = 15.5 mL). The reaction cells were photolyzed from the bottom using a 470 nm LED array constructed in our laboratory (light flux = (2.36 ± 0.05) × 10^19^ photons/min; power = 200 mW) [40]. The amount of hydrogen produced was monitored in real-time using a HY-OPTIMA™ 700 in-line process solid state hydrogen sensor from H2scan connected to the photolysis reaction cell. The sensor was calibrated by injecting known quantities of hydrogen into the photolysis cells and generating a calibration curve. The functioning of the sensor was verified by injecting a 100 μL sample from the reaction cell’s headspace into a series 580 GOW-MAC gas chromatograph equipped with a rhenium-tungsten thermal conductivity detector and a 5 Å molecular sieves column using ultra-high purity argon gas. The gas chromatograph signal was amplified with a Vernier Software instrument amplifier and recorded using Logger Pro 3.4.5 software. The gas chromatograph was calibrated for hydrogen signal sensitivity by injecting known amounts of hydrogen gas and generating a calibration curve. The reported value for hydrogen production is the average of three experiments.

## 4. Conclusions

The kinetic analysis shows that both TL and halide bound to Rh impacts observed excited state dynamics. Variation of TL and halide bound to Rh impacts rates of reactions from the formally Ru→dpp CT excited states. The ^3^MLCT states are longest lived for TL = Ph_2_phen and X = Cl and shortest for TL = phen and X = Br. The rate of intramolecular electron transfer, k_et_, to generate the ^3^MMCT state is largest when TL = phen and X = Br and smallest with TL = Ph_2_phen and X = Cl. The Φ_3MMCT_ is large in all cases varying from 0.73–0.82. Quenching of the ^3^MLCT states is very efficient and all complexes studied undergo photoinitiated electron collection to produce the Rh(I) complex. Many of these systems are known photocatalysts for H_2_O reduction to produce H_2_ with high quantum yields and turnovers with respect to known supramolecular photocatalysts. The study of the rate of quenching of the ^3^MLCT state by DMA shows rapid quenching near the diffusion control limit. Photoreduction occurs at a rate, k_q_, of (2–4) × 10^8^ M^−1^s^−1^, leading to rapid reduction of the supramolecules. This is consistent with the high thermodynamic driving force for reduction of the trimetallics by DMA which is thermodynamically favorable by 0.49–0.60 V. Analysis of the kinetic requirements for photoreduction from the ^3^MLCT and ^3^MMCT states suggests photoreduction occurs primarily from the ^3^MLCT state. These kinetic analyses provide considerable insight into the important excited state reactions of these Ru(II),Rh(III),Ru(II) supramolecular photoinitiated electron collectors, a class of molecules of interest as visible light induced photocatalysts for H_2_O reduction to H_2_.
